# Current strategies for meniscal preservation in anterior cruciate ligament reconstruction

**DOI:** 10.2340/17453674.2026.46526

**Published:** 2026-08-31

**Authors:** Håvard VISNES, Eivind INDERHAUG

**Affiliations:** 1 The Norwegian Knee Ligament Register, Department of Orthopaedic Surgery, Haukeland University Hospital, Bergen;; 2 Department of Orthopaedics, Sorlandet Hospital Kristiansand, Kristiansand; 3 Oslo Sports Trauma Research Center, Norwegian School of Sports Sciences, Oslo; 4 Sports Traumatology and Arthroscopy Research Group, University of Bergen, Bergen; 5 Haukeland University Hospital, Bergen, Norway

## Abstract

Meniscal preservation is a cornerstone of anterior cruciate ligament reconstruction (ACLR), reflecting the meniscus’ critical role in joint homeostasis and long-term knee health. Concomitant meniscal injuries are common and increasingly managed with repair rather than resection. This educational review summarizes current strategies for the most frequent acute meniscal tears encountered during ACLR. Contemporary practice favors repair whenever feasible, supported by evidence linking meniscal preservation to improved outcomes and reduced osteoarthritis risk. Lateral meniscus posterior root tears are commonly managed with transtibial or anchor-based repair. Ramp lesions require systematic inspection and selective repair, with both all-inside and suture-based techniques yielding favorable results. Bucket-handle tears are repaired using all-inside, inside-out, or combined approaches depending on tear location, with high survival rates when performed alongside ACLR. Radial tears, particularly in vascular zones, should be repaired to restore hoop stress, although optimal suture configurations remain debated. Successful outcomes rely on mechanically stable repairs, individualized rehabilitation, knee stability and alignment, and a supportive biological environment.

**Key take-home messages**

- Meniscal preservation should be prioritized whenever technically feasible during anterior cruciate ligament reconstruction (ACLR).
- Age alone should not determine reparability; tissue quality, tear morphology, vascularity, and overall joint status are more important considerations.
- Lateral meniscus posterior root tears, ramp lesions, bucket-handle tears, and radial tears should be actively identified and considered for repair.
- Successful outcomes depend on mechanically stable repair, restoration of knee stability, and individualized rehabilitation.
- Meniscal preservation is associated with improved long-term joint health and a lower risk of osteoarthritis compared with meniscal resection.

The primary goal of anterior cruciate ligament reconstruction (ACLR) is to restore knee stability and enable patients to return to their preinjury level of activity [1]. In recent years, the number of ACLRs has increased substantially in several countries, accompanied by a growing emphasis on the diagnosis and treatment of concomitant meniscal injuries. This trend towards meniscal preservation is reflected in national registries and clinical studies from several healthcare systems. In Norway, the proportion of meniscal injuries identified during primary ACLR has increased from 49% in the 2005–2010 period to 68% in the 2018–2023 period [2,3].

Data from the Norwegian Knee Ligament Register (NKLR) indicates that knee surgeons have a more proactive approach to meniscal treatment. In 2023, 71% of identified meniscal injuries were treated with repair compared with only 37% in 2013 [4]. This trend does not appear to be driven by changes in injury mechanisms or sports participation, but rather by increased awareness of the long-term consequences of meniscal damage, particularly its detrimental effects on articular cartilage. Generally, there has been a trend toward fewer meniscal resections and more repairs [5]. Long-term studies show how both medial and lateral meniscus resections independently increase the risk of osteoarthritis (OA) following ACLR [6], and systematic reviews consistently link meniscal injury at the time of ACL rupture to later knee OA [7,8]. It has further been shown how patients who have undergone meniscus repair have a lower risk of later consultation for knee OA (25–50%) compared with those who have undergone partial resection [9].

Clinical examination and MRI remain important components of meniscal tear assessment. However, no single clinical test provides sufficient diagnostic accuracy in isolation, and MRI findings should be interpreted in conjunction with clinical examination and intraoperative findings. Arthroscopy remains the reference standard for assessment of tear morphology and reparability [10].

Whether to repair a meniscus tear or not is a decision based on several factors, such as patient characteristics, meniscus tear type, and tissue quality—and the overall condition of the joint. Age alone does not significantly affect risk of failure after repair of traumatic meniscal tears in patients under 58 years [11], making it a poor predictor of reparability. However, increasing age is associated with a higher prevalence of meniscal degeneration and concomitant osteoarthritic changes. Therefore, intraoperative assessment of tissue quality, tear morphology, and vascular zone location plays a more decisive role. The viability of the tissue is best assessed by direct visual inspection and probing. It should be acknowledged that intraoperative assessment is partly subjective and depends on surgeon experience.

Advances in arthroscopic visualization, repair devices, and understanding of meniscal biomechanics and healing have expanded the range of injuries considered suitable for repair. Consequently, surgeons are increasingly attempting repair in cases that previously would have been managed with partial meniscectomy.

This review highlights the most common acute meniscal tear patterns encountered during ACLR—lateral meniscus posterior root tears (LMPRTs), ramp lesions, bucket-handle tears, and radial tears—and summarizes their current management strategies. Less frequently occurring tear patterns found at ACLR fall outside the scope of this educational review.

## Lateral meniscus posterior root tear

**Figure 1:**
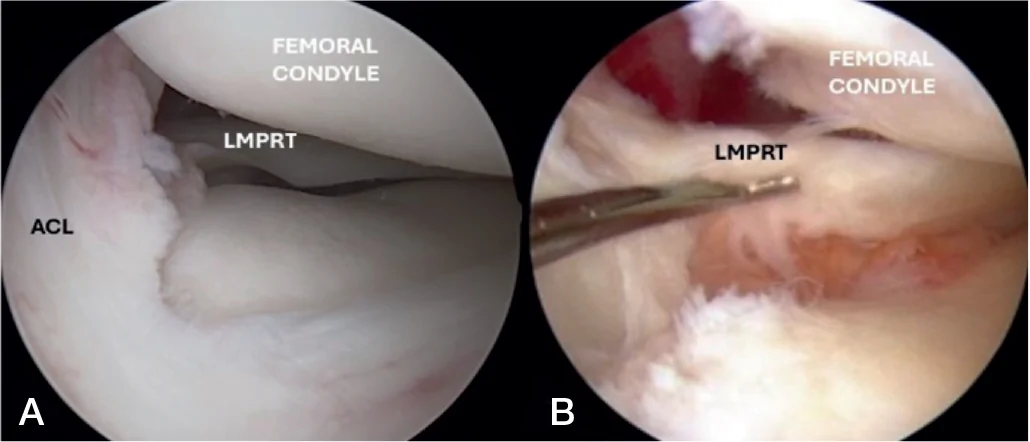
(A) A normal lateral meniscus posterior root (LMPR) in a knee with intact ACL. (B) A displaced lateral meniscus posterior root tear (LMPRT) in a knee with absent ACL

A lateral meniscus posterior root tear (LMPRT) is defined as a radial or longitudinal tear within 1 cm of the posterior root insertion, or an injury to the meniscotibial ligaments [12]. Classification systems by Laprade et al. [13] and Forkel and Petersen [14] further refined tear patterns (Figure 1).

LMPRTs are reported in 7% to 12% of knees with ACL injuries [15-17], with consistent findings across both large population-based studies and specialized case-series. Common risk factors include older age, male sex, and higher body mass index (BMI) [18]. Some studies also suggest an association with concomitant medial meniscal tears and ramp lesions [17], although findings on age remain inconsistent [19].

### Treatment strategy

According to the 2019 ESSKA consensus [11], LMPRTs should generally be repaired and are considered an indication for ACLR. Although the optimal timing remains debated, current practice in many centers is to perform surgery in the subacute phase, once range of motion has been restored, swelling has resolved, and neuromuscular control is adequate. Norwegian guidelines follow a similar approach. If the root appears stable intraoperatively (upon anterior-posterior probing with a hook from the anterior portal) and meniscotibial ligaments are intact, repair may not be necessary.

**Figure 2:**
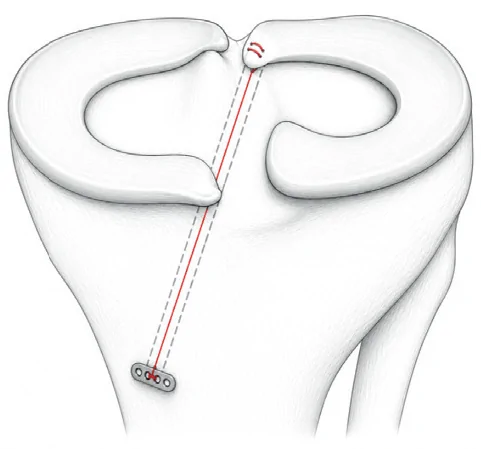
Illustration of LMPRT repair using a transtibial technique with cortical fixation over a button.

**Figure 3:**
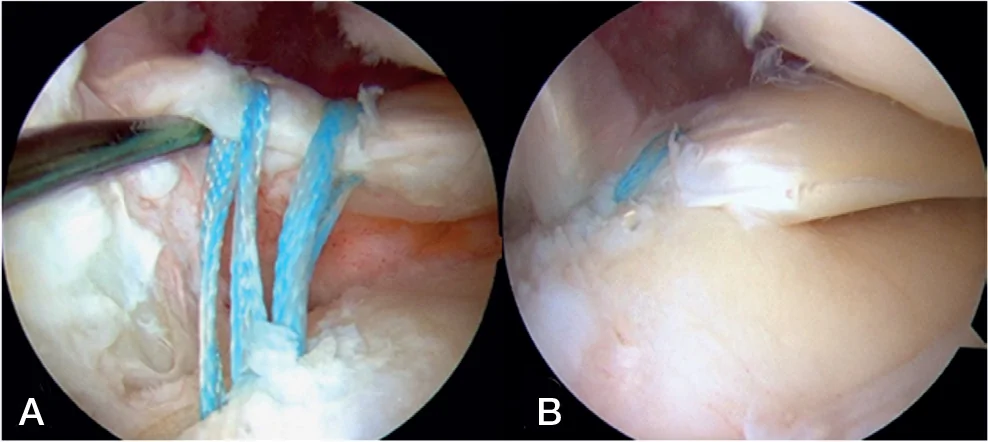
(A) Repair of an LMPRT with 2 suture tapes placed in a mattress configuration in the posterior horn of the lateral meniscus. (B) Anatomical reduction of the meniscus achieved by tensioning the transtibial sutures.

The most commonly used technique is transtibial repair, where sutures or suture-tapes are passed through the tear and fixed to the anatomical footprint via a tibial tunnel (Figures 2 and 3). In cases with poor tissue quality and/or a significant gapping of the meniscal root, a non-anatomical repair might be necessary—to restore hoop stresses and prevent over-tightening or extrusion of the meniscus. An anatomical refixation is widely supported, particularly for true root avulsions or radial tears with poor tissue [20] and has demonstrated good safety and reproducibility [21,22]. The understanding of meniscal anatomy is crucial when performing an LMPRT repair. A separate drill tunnel for the repair needs to be placed posterior to and separate from the ACL tunnel—with interspaced entry points at the anterior tibia. Care should be taken to avoid tunnel convergence. The emergence of low-profile drill guides has simplified tunnel placement, further improving the reproducibility of surgery. However, technical aspects—such as single vs double tunnels, insertion site preparation, and suture configuration—remain debated [23–25].

Suture anchor repair is an alternative, avoiding tibial tunnels but requiring greater technical expertise. It may be particularly useful in selected radial tears with sufficient remaining tissue allowing side-to-side repair [16,20,22,23]. Hybrid techniques combining anchor-based and transtibial fixation may offer advantages in complex cases.

Overall, LMPRT repair performed alongside ACLR yields favorable outcomes [26, 27], reporting healing rates exceeding 80% [28].

## Ramp lesions

A ramp lesion is a tear of the peripheral attachment of the posterior horn of the medial meniscus and is closely associated with ACL injury [29]. Several subclassification systems have been proposed [30-32], but none are widely used or validated in large-scale studies [33].

**Figure 4:**
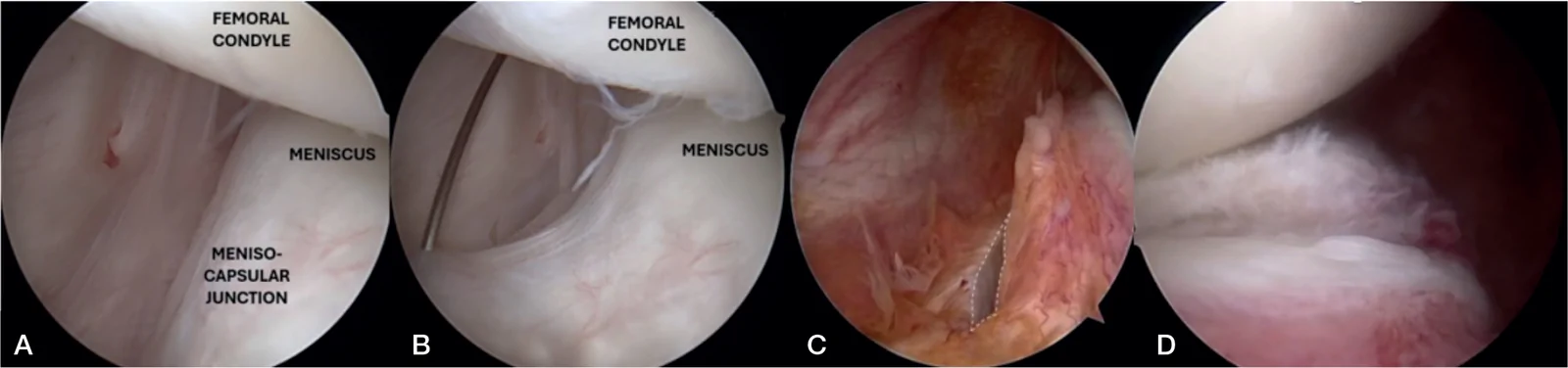
(A) Top-down illustration of a ramp lesion in the posterior part of the medial meniscus. (B) Arthroscopic view of ramp is seen (C) A tear of the meniscocapsular ramp is outlined (white dotted line). (D) Ramp lesion seen from the posterior medial portal.

The reported prevalence of ramp lesions during ACLR varies widely from 9% to 42% in published series [34-37], whereas a national registry study reported a lower rate of 7.8% [3]. This discrepancy likely reflects inherent differences in study design and detection methods [30,35,37-39]. Ramp lesions should be actively sought in all ACL-injured knees. Routine arthroscopic evaluation should include trans-notch visualization (the Gillquist view) and probing of the posterior horn of the medial meniscus. Without this, “hidden” ramp lesions may be missed (Figure 4). Time from injury to surgery may also influence detection, as some lesions may partially (or fully) heal before ACLR [40].

### Treatment strategy

Management of ramp lesions remains controversial, with no uniform treatment recommendation [41]. Current consensus suggests careful inspection in all ACLRs and repair of unstable lesions based on biomechanical considerations [42].

For small, stable lesions, nonoperative strategies—such as leaving the lesion untreated or performing trephination—may be sufficient. A randomized controlled trial by Liu et al. [40] found similar outcomes between repair and trephination alone for stable (small-to-medium lesions) treated during ACLR.

Among surgical options, the all-inside technique is most commonly used employing implant-based devices to repair the meniscocapsular junction. During this procedure, it is crucial to extend the knee to reduce the capsular separation before deploying the implant, thereby restoring anatomical alignment of the meniscocapsular complex (see Figure 4A) [43].

**Figure 5:**
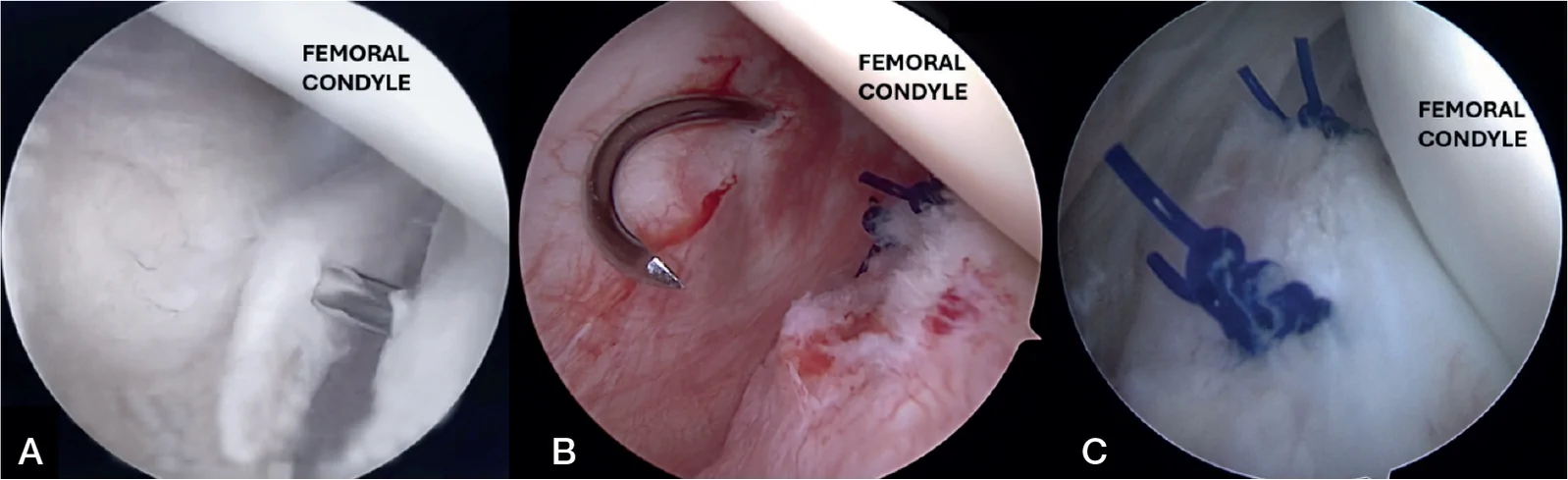
(A) If an all-inside repair (from anterior) is attempted, the knee must be held in extension to reposition the meniscocapsular lesion. (B) Suture from a posteromedial portal with a suture hook is done with the knee in 90 degrees of flexion (C) A completed repair of the meniscocapsular junction (meniscal ramp) with absorbable sutures.

**Figure 6:**
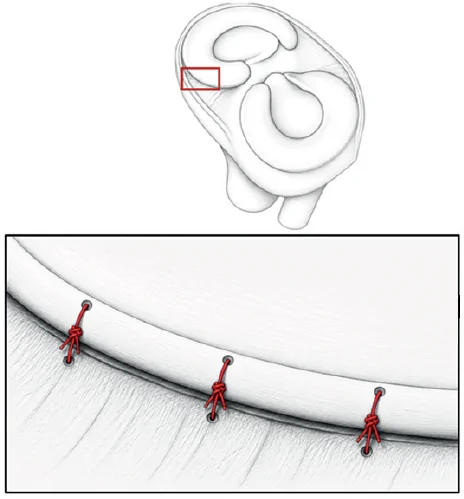
Schematic illustration of repair of a medial meniscal ramp lesion through a posteromedial portal.

Alternative techniques include hook repair through a posteromedial portal allowing direct visualization and suturing of the lesion (Figures 5 and 6) and the inside- out technique [44], which provides strong fixation with precise suture placement, particularly in larger or more peripheral tears. For the suture hook repair technique, enhanced vision can be gained by using a 70-degree arthroscope instead of the standard 30-degree scope—or by using an accessory posteromedial viewing portal. Although more technically demanding, both approaches remain valuable depending on surgeon preference and tear characteristics.

Overall, ramp lesion repair performed alongside ACLR yields favorable outcomes. Studies report significant improvements in knee function [41,45], with healing rates exceeding 80% [46]. However, these findings should be interpreted with caution, as the reported healing rates are largely based on selected cohorts undergoing second-look arthroscopy, and the reliability of MRI for detecting and assessing healing of ramp lesions remains limited.

## Bucket-handle tears

**Figure 7:**
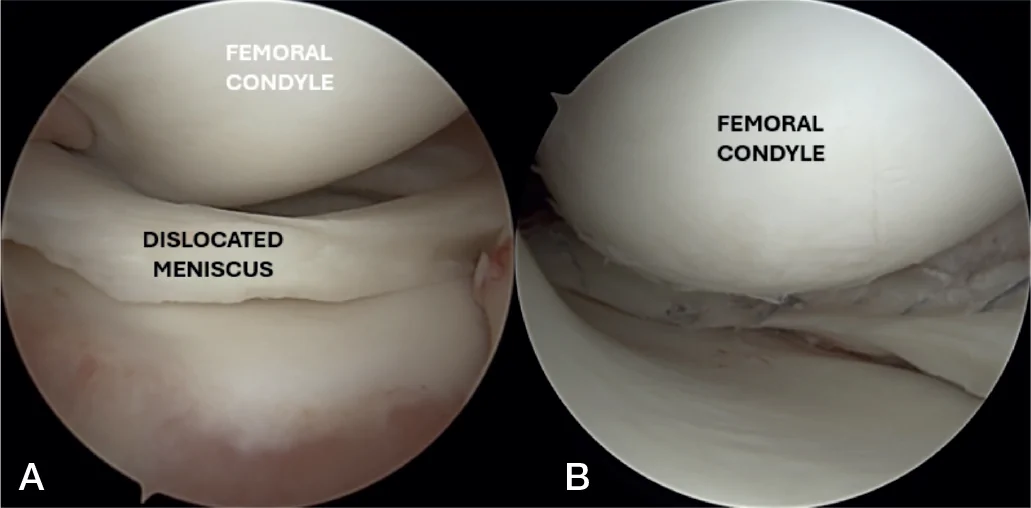
(A) Anteriorly dislocated bucket-handle tear resulting in a clinically “locked knee” with extension deficit. (B) Repositioned and repaired bucket-handle tear with multiple vertical all-inside sutures.

Bucket-handle meniscal tears (BHMTs) are full-thickness longitudinal tears in which the anterior and posterior attachments remain intact [47]. Typically, the central fragment displaces into the intercondylar notch, often causing mechanical locking and extension deficit (Figure 7) [48].

BHMTs account for 9% to 26% of all meniscus tears [49, 50]. In the setting of ACLR, registry data report a prevalence of 18.5% [3], while other series identified bucket-handle tears as the most common meniscal injury, occurring in about 14% of cases [51].

### Treatment strategy

Repair is recommended for unstable tears, including bucket-handle configurations [11, 50]. In acute cases with a locked knee, early surgery—ideally within the first few weeks—is advised to restore motion and prevent neuromuscular dysfunction [11]. Notably, even chronic bucket-handle tears can achieve good outcomes when repaired concurrently with ACLR [52]. The ESSKA meniscus consensus does not define a strict age limit for meniscal repair. Instead, it recommends that reparability be determined by tear morphology, tissue quality, and vascularity. Although successful repairs have been reported in older patients, chronological age alone should not be used as a contraindication [11] and repair is now the standard of care whenever feasible.

**Figure 8:**
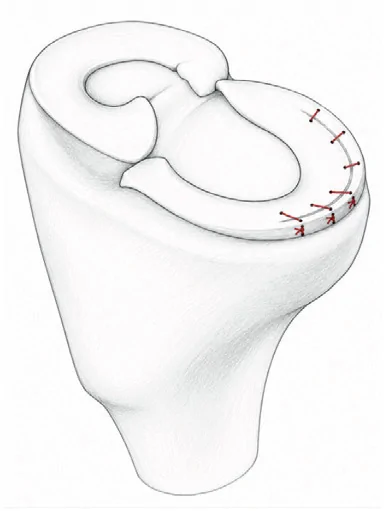
Illustration of medial bucket-handle hybrid repair with 3 all-inside sutures (posterior) and 3 inside-out.

**Figure 9:**
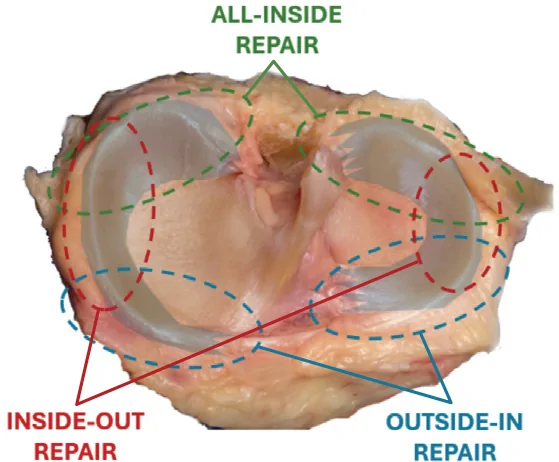
Zone-specific repair of bucket-handle tears depending on the extent of rupture. Often, a combination of techniques should be applied for an optimal result.

The inside-out technique has traditionally been considered the gold standard [53-55]. However, the all-inside repair has become increasingly popular due to its technical simplicity, shorter operative times, and reduced risk of neurovascular complications (Figures 7 and 8) [56-58]. Evidence comparing techniques is mixed: some studies report higher failure rates for medial repair using all-inside devices [59] while others show comparable healing and complication rates, with added efficiency and safety benefits [57]. In practice, repair strategy is often tailored to tear location: all-inside technique for posterior tears, combined all-inside or inside-out approaches for mid-body tears, and outside-in repair for anterior horn lesions (Figure 9). For lateral meniscus tears—especially near the anterior–middle junction—all-inside repair is often preferred due to the risk of peroneal nerve injury with inside-out techniques [60]. No clear consensus exists for comparable medial tears.

Overall, outcomes are favorable when BHMT repair is performed alongside ACLR, with reported survival rates of 80% to 95% [61-63]. Current repair strategies also reduce reoperation rates compared with stage procedures [64, 65], although failure rates of up to 29% [66] have been reported in some series using all-inside fixation.

## Radial tears

**Figure 10:**
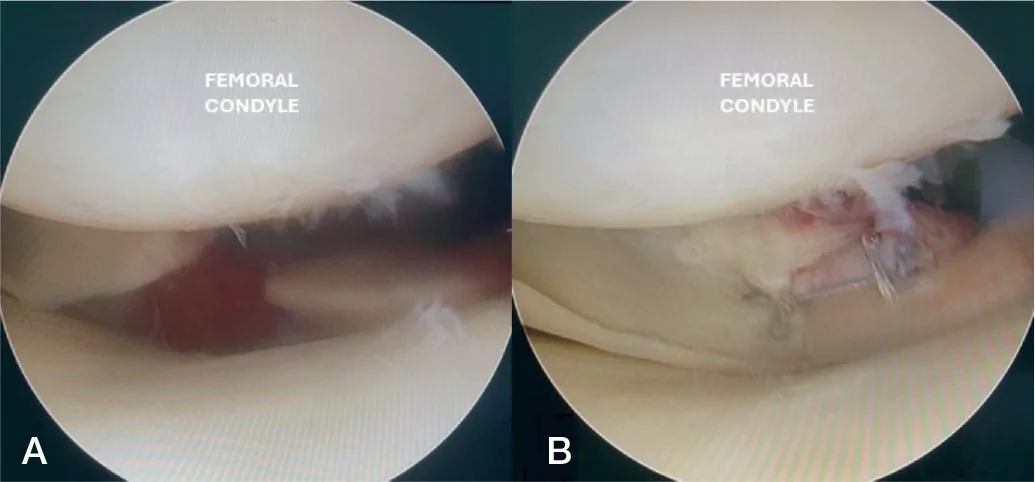
(A) A posterior medial complete radial meniscal tear with significant gapping. (B) Repair of the meniscus root with all-inside hash-tag suture configuration.

Radial tears are biomechanically significant because they compromise the ability of the meniscus to transmit hoop stress, thereby increasing tibiofemoral contact pressure and reducing its load-sharing function [67,68]. While even partial radial defects may alter load distribution, tears involving 60% to 90% of the meniscal width—especially those extending to the peripheral vascular rim (Figures 10) -– are most consistently associated with a contact mechanics similar to total meniscectomy. The exact threshold for critical loss of tensile integrity remains unclear and likely depends on tear location, morphology, and tissue quality [67–69].

The reported incidence of radial meniscus tears varies, partly due to inconsistent definitions and inclusion of root tears. However, arthroscopic studies suggest a prevalence of approximately 5% to 15% [11,70].

Radial tears in Zones 1 and 2 (“red–red” and “red–white”) should be repaired to preserve circumferential fiber integrity, whereas partial meniscectomy is typically reserved for small lesions in the avascular Zone 3 (“white–white”) [11,67]. Given their biomechanical impact, early repair is recommended to prevent progression to complete disruption and irreversible loss of hoop function.

**Figure 11:**
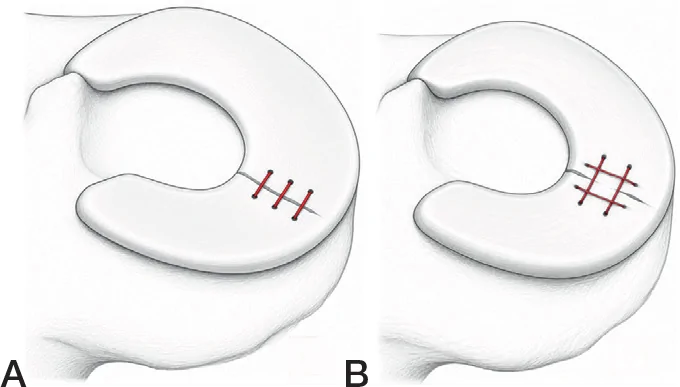
Illustration of (A) side-to-side suture and (B) vertical rip-stop sutures in a radial rupture.

**Figure 12:**
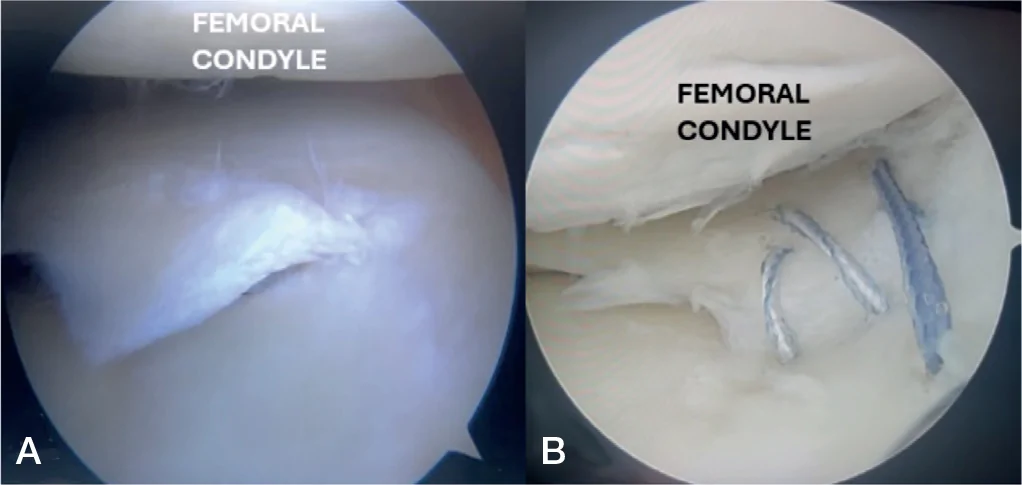
(A) A lateral meniscus radial tear extending beyond 2/3 of the meniscal with (B) repair of the meniscal tear using inside-out technique with 3 horizontal sutures.

Multiple arthroscopic techniques are used, most commonly inside-out rebar and all-inside approaches. No single suture configuration has proven superior, but the “hashtag” (cross-suture) construct—combining side-to-side and vertical rip-stop sutures like the suture—is widely used to restore tension and resist tear propagation (Figures 11 and 12) [71]. A transtibial pullout technique may also be used to improve stability and reduce meniscal extrusion [72].

Repair of radial tears generally yields favorable outcomes, with good patient-reported results and low failure rates [73]. Second-look arthroscopy studies report healing rates ranging from 60 to 86%, with no clear superiority between techniques or suture configurations [73,74].

## Fundamental principles and key factors in rehabilitation after ACLR and meniscus injuries

**Figure 13:**
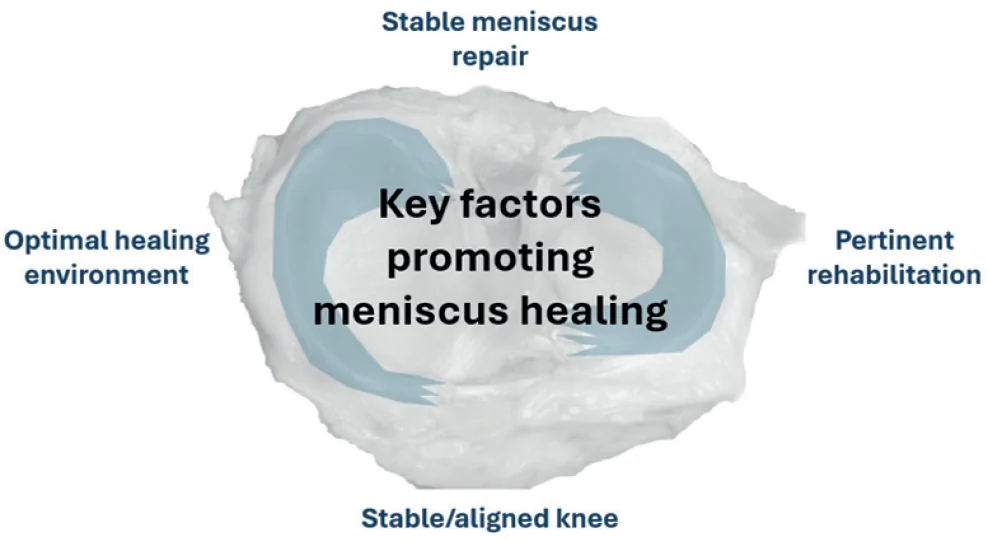
Fundamental principles for optimal outcomes after meniscus preservation in ACL surgery.

Achieving an optimal functional outcome after combined ACLR and meniscus surgery relies on 4 fundamental principles (Figure 13):

### 1. Mechanically stable meniscus repair

A stable repair is essential for successful healing. This requires anatomic reduction, secure fixation tailored to tear type and tissue quality, and preservation or restoration of hoop stress. Without meniscal stability, biological healing and functional recovery are compromised

### 2. Appropriately tailored postoperative management and rehabilitation

Structured, active rehabilitation is central to recovery after combined ACLR and meniscal surgery, with the overall goal of restoring knee function and enabling a safe return to activity. While rehabilitation after isolated ACLR is relatively well described, evidence guiding rehabilitation after meniscal repair remains limited and no universally accepted protocol exists [75-77]. Consequently, postoperative management should be individualized according to tear morphology, repair stability, and the biomechanical consequences of loading.

A key principle is whether circumferential hoop stress is preserved. Stable longitudinal tears generally tolerate early weightbearing because axial loading compresses the repair site, whereas radial tears and lateral meniscus posterior root tears disrupt hoop integrity and may experience distracting forces during loading. For this reason, more protective rehabilitation strategies are commonly recommended for unstable tears, often including restricted weightbearing for 4–6 weeks and limitation of loaded knee flexion beyond 90° during the early healing phase. In contrast, repairs of stable longitudinal tears are frequently managed with earlier weightbearing progression and less restrictive protocols.

Although accelerated rehabilitation protocols have shown acceptable outcomes in selected meniscal repairs, excessive early loading, deep flexion, pivoting, and impact activities may compromise healing in complex repairs. Rehabilitation should therefore balance biological protection of the repair with prevention of stiffness, quadriceps inhibition, and persistent neuromuscular deficits. Progression is preferably criterion-based rather than strictly time-based, with return to pivoting sports typically delayed for several months depending on tear type, healing, strength recovery, and functional performance.

### 3. A stable, well-aligned knee joint

Normal knee kinematics are critical to protect both the ACL graft and the meniscal repair. This requires a well-positioned, stable ACLR without residual ligamentous laxity. Malalignment (varus/valgus) or increased tibial slope can overload the meniscus and impair healing, particularly in root tears. While corrective osteotomy is rarely indicated in primary ACLR, it may be considered in selected revision cases.

**Table:** Clinical rehabilitation guidelines based on meniscal tear type in anterior cruciate ligament reconstruction. Rehabilitation should be individualized based on tear morphology, repair stability, and proximity to the meniscal root

Tear type Hoop stress integrity	Weightbearing	ROM	Key precautions
Lateral meniscus posterior root tear			
Functionally absent	Partial (4–6 weeks)	0–90° early	Avoid axial loading in flexion, rotational/shear stress, and hamstring activation in deep flexion; delay progression of loading
Ramp lesions			
Partially preserved	As tolerated	Full as tolerated (if stable repair)	Avoid combined flexion–rotation loading, excessive tibial internal rotation, and loaded terminal flexion early
Bucket-handle tears			
Restored if anatomically reduced and stable fixation	As tolerated (if stable fixation)	Early (typically 0–90° if repaired)	Avoid deep knee flexion, pivoting/twisting, and high shear forces until healing is established
Radial tears			
Disrupted (extent dependent; greater near root)	Partial (4–6 weeks)	Full; avoid loaded early flexion >90°	Delay strengthening and return to pivoting; avoid axial loading in deep knee flexion

**Meniscal preservation versus resection: practical considerations**

*Factors favoring meniscal preservation*

- Good tissue quality
- Acute traumatic tears
- Repairable tear morphology
- Tears located in vascular zones
- Concomitant ACL reconstruction
- Young and active patients

*Factors favoring partial resection*

- Irreparable tear morphology
- Poor tissue quality
- Advanced meniscal degeneration
- Avascular tear location
- Established osteoarthritic changes
- Failed previous repair with non-repairable tissue

*Key principle*

 When technically feasible, preservation of meniscal tissue should be preferred over resection. Age alone should not be considered a contraindication to meniscal repair.

### 4. Favorable biological environment

Meniscal healing depends on a supportive biological environment. Healing rates are higher when repair is performed concurrently with ACLR compared with isolated meniscal repair [78-80]. This may relate to biological factors such as intra-articular bleeding, marrow stimulation from tunnel drilling, and growth factor release, although the exact mechanisms remain uncertain. In contrast, adjunctive therapies such as platelet-rich plasma or intra-articular blood clots currently have limited and unclear benefit.

## Conclusion and future directions

Meniscal preservation has become a cornerstone of modern ACLR, reflecting a paradigm shift toward joint preservation and potentially better long-term knee health. This review underscores the growing recognition and surgical management of acute complex meniscal injuries—such as lateral meniscus posterior root tears, ramp lesions, bucket-handle tears, and radial tears—at the time of ACLR. Advances in arthroscopic techniques and instrumentation have broadened repair indications, supported by accumulating evidence of favorable healing rates and improved patient-reported outcomes.

Nevertheless, optimal repair strategies remain debated, particularly regarding suture configuration, timing, and technique selection based on tear morphology and location. Postoperative rehabilitation protocols, including weightbearing and range-of-motion restrictions, also remain uncertain due to persistent knowledge gaps. Future research should prioritize large-scale, prospective, and registry-based studies to establish the long-term benefits of meniscal repair in ACLR. Emerging innovations in biologic augmentation and advanced imaging hold promise for enhancing both healing potential and surgical precision. Throughout these advances, one guiding principle remains: save the meniscus whenever possible.
